# Reduced representation bisulphite sequencing of ten bovine somatic tissues reveals DNA methylation patterns and their impacts on gene expression

**DOI:** 10.1186/s12864-016-3116-1

**Published:** 2016-10-06

**Authors:** Yang Zhou, Lingyang Xu, Derek M. Bickhart, El Hamidi abdel Hay, Steven G. Schroeder, Erin E. Connor, Leeson J. Alexander, Tad S. Sonstegard, Curtis P. Van Tassell, Hong Chen, George E. Liu

**Affiliations:** 1 Shaanxi Key Laboratory of Agricultural Molecular Biology, College of Animal Science and Technology, Northwest A&F University, Yangling, Shaanxi 712100 People’s Republic of China; 2 Animal Genomics and Improvement Laboratory, BARC, USDA-ARS, Building 306, Room 111, BARC-East, Beltsville, MD 20705 USA; 3 Institute of Animal Science, Chinese Academy of Agricultural Science, Beijing, 100193 People’s Republic of China; 4 USDA Agricultural Research Service, Fort Keogh Livestock and Range Research Laboratory, Miles City, MT 59301 USA; 5 Recombinetics, Inc., St. Paul, MN 55108 USA

**Keywords:** Cattle genome, Somatic tissue, DNA methylation, RRBS (reduced representation bisulphite sequencing)

## Abstract

**Background:**

As a major epigenetic component, DNA methylation plays important functions in individual development and various diseases. DNA methylation has been well studied in human and model organisms, but only limited data exist in economically important animals like cattle.

**Results:**

Using reduced representation bisulphite sequencing (RRBS), we obtained single-base-resolution maps of bovine DNA methylation from ten somatic tissues. In total, we evaluated 1,868,049 cytosines in CG-enriched regions. While we found slightly low methylation levels (29.87 to 38.06 %) in cattle, the methylation contexts (CGs and non-CGs) of cattle showed similar methylation patterns to other species. Non-CG methylation was detected but methylation levels in somatic tissues were significantly lower than in pluripotent cells. To study the potential function of the methylation, we detected 10,794 differentially methylated cytosines (DMCs) and 836 differentially methylated CG islands (DMIs). Further analyses in the same tissues revealed many DMCs (including non-CGs) and DMIs, which were highly correlated with the expression of genes involved in tissue development.

**Conclusions:**

In summary, our study provides a baseline dataset and essential information for DNA methylation profiles of cattle.

**Electronic supplementary material:**

The online version of this article (doi:10.1186/s12864-016-3116-1) contains supplementary material, which is available to authorized users.

## Background

DNA methylation has been widely recognized as a regulatory epigenetic mechanism that is crucial for cellular reprogramming, tissue differentiation and normal development [1–5]. Aberrant methylation patterns may lead to numerous diseases [6, 7]. However, to date, DNA methylation patterns have been well characterized in only a few species, including Arabidopsis, human and rodents [8–13]. Moreover, different methylation mechanisms have been proposed for mammals versus plants [14]. Unlike plants, DNA methylation in mammals almost exclusively occurs in the CG context while DNA methylation in the non-CG context was thought to be nearly absent in somatic tissues except for pluripotent stem cells, brain and oocytes [1, 10, 15, 16]. Only a few human and rodent studies have focused on non-CG methylation in germline cells [11, 16–19]. Recently, epigenome maps of the human body showed unexpected presence of non-CG methylation in all somatic tissues [11]. However, the functional aspects of this methylation are not yet well understood. Mammalian DNA methylation patterns were thought to be initiated by *de novo* DNA methyltransferases DNMT3a/3b and maintained by DNMT1 during DNA replication [20, 21]. However, this “two step” model does not explain non-CG methylation beyond the symmetric context of CG methylation [22]. Moreover, demethylation mechanisms have been reported to be different between the CG and non-CG context [14]. Thus, CG and non-CG methylation have been thought to undergo different mechanisms [22].

Our knowledge of DNA methylation pattern in livestock, even for CG context, is still limited when compared to humans and rodents. A few genome-wide DNA methylation studies were reported with limited tissue types and low resolution in cattle, pigs, sheep and horses [23–28]. Two studies reported the genome-wide methylation of several pig tissues at single-base resolution using the reduced representation bisulfite sequencing (RRBS) method [29, 30]. In cattle, we found a couple of studies for placental and muscle tissues using methylated DNA immunoprecipitation combined with high-throughput sequencing (MeDIP-seq) which did not provide a single-base resolution [23, 24, 31]. Recently, an evolutionary analysis of gene body DNA methylation patterns was reported in mammalian placentas using whole genome bisulfite sequencing (WGBS) [32]. However, for cattle samples, due to their low genome coverage (up to 1.25×), this study only offered a coarse resolution instead of a single-base resolution. Therefore, knowledge of how DNA methylation affects gene expression, phenotype, animal health and production is urgently needed. In line with the Functional Annotation of Animal Genome (FAANG) project [33], the present study is an important step towards understanding DNA methylation patterns and their functions.

RRBS is an effective method to describe the methylation patterning on a genome-wide level [34]. Unlike MeDIP-seq and methyl-binding domain sequencing (MBD-seq), RRBS can detect methylation in a single-base resolution including information about all three methylation contexts (CG, CHG and CHH). On the other hand, WGBS is the most comprehensive method for describing DNA methylation. Compared to the high cost of WGBS, RRBS enriches for high CG regions, which range from 5.3 % in zebrafish 8.3 % in pig of total genome CG sites, and has been proven as a less expensive method to study DNA methylation in the presumed functionally most important part of a genome [29].

Here, we constructed the genome methylation profiles of ten diverse tissues of cattle using the RRBS method. We describe the landscapes of the DNA methylome and common methylation patterns among the tissues. To assess non-CG methylations, we compared distributions between the somatic tissues and published WGBS data of bovine oocytes [32]. We further studied differential methylation, which may be involved in tissue development, by detecting differentially methylated cytosines (DMCs) and differentially methylated CG islands (DMIs) and comparing methylation levels among these tissues. By combining RNA-Seq data from the same tissues, we detected many DMCs and DMIs that may affect tissue development through regulating gene expression. This study supplies essential information on the cattle methylome and provides a reference dataset for further study of DNA methylation.

## Results

### Assessment of the RRBS data

**Fig. 1:**
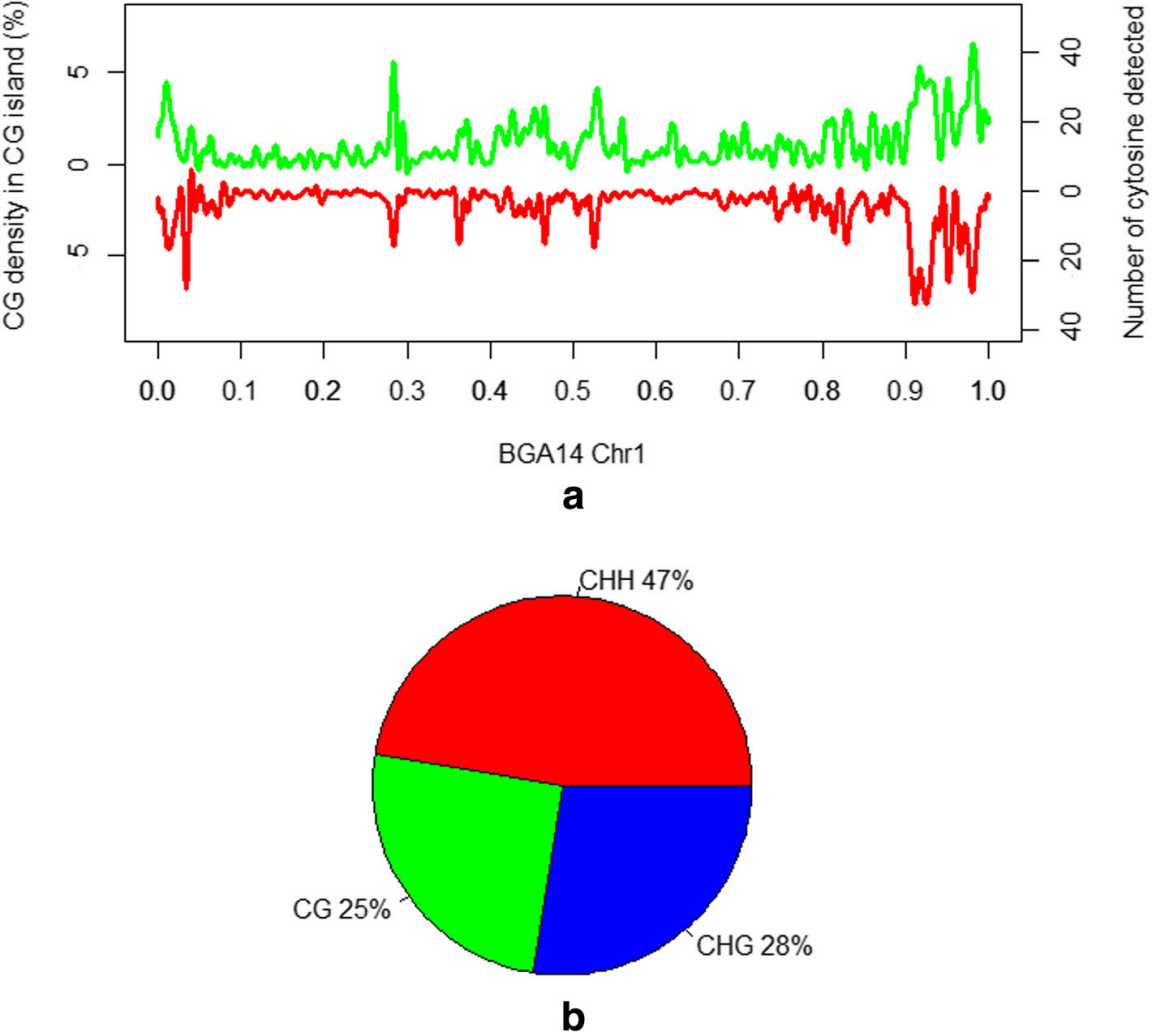
Chromosomal distribution and context percentage of detected cytosines. **a** The density distribution of cytosines on chr1 using 20-Kb non-overlapping windows. The *green line* represents the density distribution of CG in the CG island calculated using the UMD3.1 bovine reference genome assembly; and the *red line* represents the density distribution of cytosines detected in the BGA14 (testis) on chr1. **b** The fraction of cytosines within different contexts detected by RRBS for all ten tissues

To characterize DNA methylation patterns in cattle, we applied RRBS analysis for ten different tissues (Additional file 1: Table S1) from the Hereford cow L1 Dominette 01449 and her progeny/relatives. Dominette was the cow whose genome was sequenced to construct the cattle genome reference assembly [35, 36]. The ten tissues were chosen from the previous Bovine Gene Altas study [37]. They were distributed in different simplex clusters and spanned different development stages and physiological periods. A total of ten libraries were constructed with 150–400 bp DNA fragments and each produced a minimum of 3 Gb clean reads, an average of 41 % of which were uniquely mapped to the cattle reference assembly (UMD3.1). To guarantee the quality and quantity for each cytosines at the same time, we first selected the threshold we would use to filter cytosines with low confidence. The common shared cytosines with less than 0.2 standard deviations from the average methylation level among the ten samples were selected for cluster analysis at different filtering thresholds (3 to 10 × coverage). The cluster results became stable after removing cytosines with coverage below 8 ×. Moreover, the cytosines with 8 × coverage distributed almost same as the cytosines above 8 ×, indicating the influence of low-coverage cytosines was suppressed (Additional file 2: Figure S1). Thus, only the cytosines with at least eight reads were considered for further study. RRBS is known to enrich for high CG density regions of the genome. In our study, the distribution of the detected cytosine number per 20 Kb was consistent with that of the CG density on the genome (Fig. 1a). Totally, we obtained 1,868,049 cytosines in the CG-enriched region throughout the whole genome for further study. The relative prevalence of each sequence context detected throughout the genome was assessed, revealing that 25 % were in the CG context, 28 % were in the CHG context and 47 % were in the CHH context (Fig. 1b). This result illustrates that there were a considerable number of cytosines located in a non-CG context captured by the RRBS method. We further validated 19 randomly selected CG sites in four regions using four tissues and achieved a 62 % success rate, which is defined as CG with methylation level difference less than 0.2 between RRBS and bisulfite PCR sequencing results (Additional file 1: Table S2).

### Global DNA methylation in diverse cattle tissues

The methylation profiles of different contexts in cattle were consistent with other species. The ten bovine somatic tissues showed similar global methylation, with Pearson’s correlation scores ranging from 0.93 to 0.98. While in the pig study [29], closely related tissues were used and yielded slightly higher Pearson’s correlations (>0.95). The CGs were either enriched at a low methylation level (<20 %) or high methylation level (>80 %), while both non-CG contexts were enriched only at a low methylation level (Additional file 2: Figure S2a, b, c). Totally, we observed average genome-wide levels of 33.5 % CG, 1.1 % CHG and 1.5 % CHH methylation in CG-enriched regions. The CG methylation levels ranged from 29.87 to 38.06 % among different tissues (Table 1). Unexpectedly, we did not detect a significantly higher non-CG methylation level in the frontal cortex, which in the adult stage generally is greater than in other tissues [38]. One explanation was that our frontal cortex sample was collected from a juvenile stage.

**Table 1:** Sequencing and mapping summary

Tissue ID	Tissue name	Clean reads	Unique mapped reads	Unique mapping rate (%)	CG methylation (%)	Non-CG methylation (%)	Bisulfite conversion rate (%)
BGA13	Skeletal muscle near ceasarian	62,431,346	27,615,411	44.23	33.87	1.45	99.38
BGA14	Whole testes^a^	65,883,038	23,323,753	35.40	37.00	0.94	99.45
BGA19	Mammary/parenchyma^a^	61,978,584	27,862,415	44.95	30.50	1.31	99.28
BGA22	Uterus intercaruncular^a^	62,431,548	26,830,069	42.98	33.22	1.45	99.41
BGA47	Frontal cortex^a^	63,601,202	22,808,676	35.86	30.89	1.48	99.30
BGA60	Abomasum^a^	62,173,874	28,496,274	45.83	38.06	1.04	99.25
BGA62	Ileum^a^	65,228,026	23,666,863	36.28	33.54	1.50	99.04
BGA81	Rumen^a^	62,646,332	25,923,247	41.38	29.87	1.44	99.28
BGA135	Nucleated blood cells^a^	63,611,924	23,184,841	36.45	36.03	1.54	99.07
BGA173	D 90 lactating mammary gland	62,474,748	28,581,463	45.75	32.04	1.36	99.33

^a^Tissues with RNA-seq data

### Comparison of the CG and non-CG methylation patterns in cattle somatic tissues

**Fig. 2:**
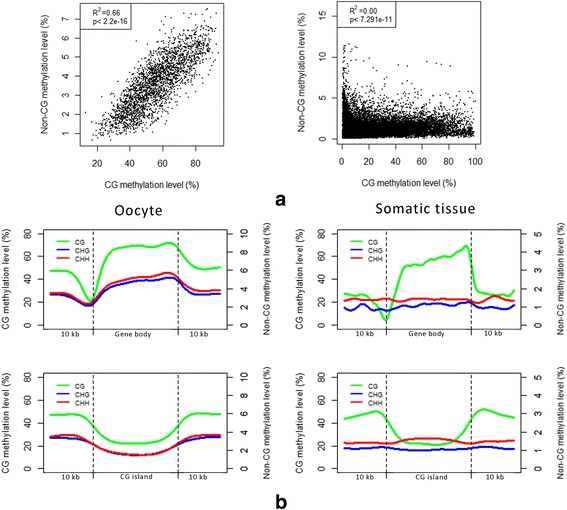
Different methylation patterns between oocyte and somatic tissues in cattle. **a** Correlation analysis of CG and non-CG methylation using 1-Mb non-overlapping windows. **b** Methylation distributions of the three methylation contexts in genic regions and CG islands. Note: all figures for somatic tissues were from the merged data after examining results individually that did not show differences between them

In mouse oocytes, non-CG methylation showed high correlation with CG methylation at the genome-wide level and was enriched in high CG regions [16]. We confirmed this correlation between CG and non-CG methylation in bovine oocytes using WGBS data downloaded from a recent publication [32] (Fig. 2a, Additional file 2: Figure S3). However, within the dataset obtained from bovine somatic tissues, we did not detect significant correlation between them. This may indicate that non-CG methylation levels were too low to measure reliably in somatic tissues as compared to oocytes (Fig. 2a).

To better understand the methylation patterns of CG and non-CG contexts in this study, we first annotated the cytosines within different genomic structures or features. For example, we detected not only the cytosines present in the nuclear genome but also the cytosines in the mitochondrial genome and the unplaced sequences (chrUn) (Additional file 2: Figure S4). DNA methylation in the mitochondrial genome was extremely low. On the other hand, both the CG context and CHG context showed the highest methylation level on chrUn. This is consistent with the notion that chrUn contains the sequences which cannot be placed on the chromosomes due to their repetitive nature, and high DNA methylation can help to repress those repeats to maintain genome stability and integrity [39, 40]. Further methylation analysis of repeat elements supported this observation. But the three methylation contexts appeared to have different distributions on different repeat elements (Additional file 2: Figure S5). Among the tested repeat elements, CG methylation was the most abundant while the non-CG methylation was lowest in SINEs. Additionally, we examined the methylation levels separately within the ± 10 Kb windows around the genic regions and the CG islands (Fig. 2b, Additional file 2: Figure S6). The CG methylation displayed the same patterns near the genic regions and the CG islands between oocytes and somatic tissues. Around the genic regions, the CG methylation level was lowest immediately upstream of the transcription start site (TSS) and increased towards the end of the last exon. Within the CG islands, the CG methylation level was lower than the level in the neighboring regions. On the contrary, when we compared oocytes to somatic tissues, non-CG methylation displayed different patterns near the genic regions and the CG islands. In oocytes, the patterns of non-CG methylation were similar to those of CG methylation in both genic regions and CG islands. However, in somatic tissues, overall non-CG methylation was decreased to almost the same level as the TSS. For somatic tissues, we did not observe large changes for the non-CG methylation either in the genic regions or CG islands.

RRBS allowed us to assess single-base methylation events in a region, which made it possible to evaluate the relationship between the methylation levels of adjacent cytosines. We examined the correlation between methylation patterns at adjacent cytosines using an autocorrelation method among different sequence contexts in ten somatic tissues (Additional file 2: Figure S7). In Arabidopsis, positive correlations were found between the two strands in both the CG contexts and the non-CG contexts [41]. In this study, we found highly positive correlations between the methylation levels of adjacent CGs on either same or different strands. The correlation level decreased as the distance increased between the two CGs, but its *R* value was still greater than 0.8 as the distance reached over 40 bp. This was probably a reflection of regional foci of methylation for the CG context [8]. Large differences were detected for the non-CG contexts where we saw a medium correlation (*R* = 0.7) for the two cytosines in the two neighboring CHH (or CHHCHH) motifs on one sense strand, and a further decreased correlation as the distance increased. Moreover, we did not observe high correlations across the different contexts.

### Characterization of CG island methylation

The CG island has been described as one of the most important methylation features of the genome. It was thought to be methylated differently from the non-CG island region in mammals [42]. In CG islands, CGs usually remain unmethylated or lowly methylated while in the non-CG island regions, CGs are heavily methylated. In cattle somatic tissues, the average methylation level of CG in non-CG islands was 72.02 % while that in CG islands was 24.22 %, which was lower than the average methylation (51.59 %) of CG at CG island shores (Additional file 2: Figure S7a). However, there were still 13 % of CG islands which had a methylation level over 80 % (Additional file 2: Figure S8b). It is noted that this uneven distribution might also be related to the bias of RRBS, as the CG density normally is high near both centromeric and telomeric regions. To decrease the effects of tissue differences and the RRBS method, we selected 3761 CG islands within less than 0.2 standard deviations of the average methylation level among the ten samples and calculated their average methylation levels in non-overlapped windows of 10 % length of the corresponding chromosome. The results showed that the average methylation levels of CG islands within both terminal windows were higher than other internal windows (Additional file 2: Figure S8c). The chromosome ends like telomeres were known to be enriched for telomere repeats, whose methylations were thought to be related to telomerase activity [43]. The adjacent subtelomeric regions were enriched with a high density of CG sequences and high methylation levels. We suspect that the highly methylated CG islands may be involved in controlling genome terminal stability.

### Identification of differentially methylated cytosines (DMCs) and differentially methylated CG islands (DMIs) related to gene expression

**Fig. 3:**
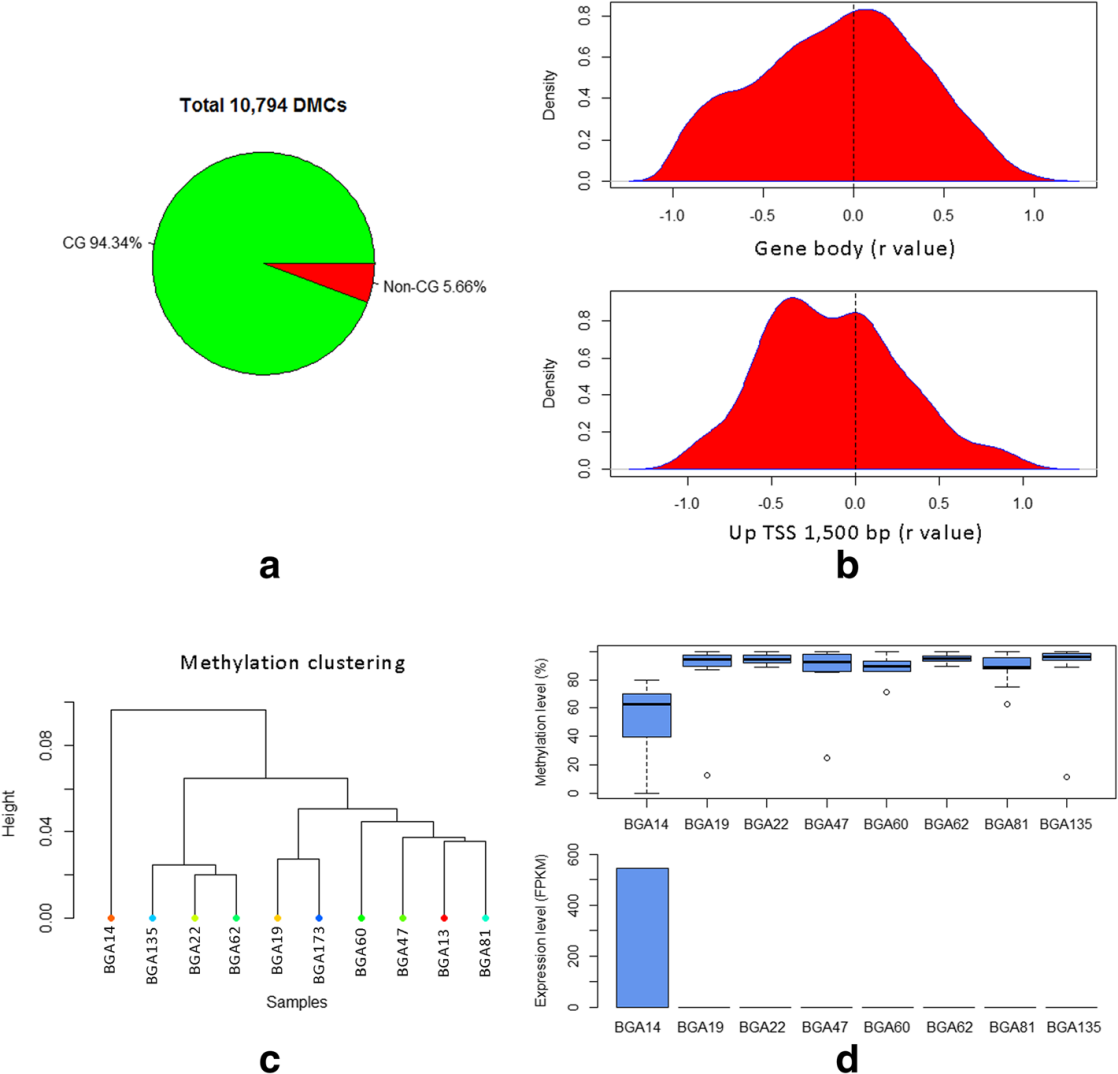
Analysis of different methylated cytosines (DMCs) and differential methylated CG islands (DMIs). **a** Fractions of DMCs in the CG and non-CG contexts. **b** Correlation between CG methylation and gene expression in the regions of 1500 bp upstream of the TSS and gene bodies. **c** Hierarchical cluster analysis for different tissues by methylation level. **d** The effect of DMI methylation on bta-mir-202 expression, top: methylation distribution of CGs in DMIs by tissue, bottom: expression level of bta-mir-202 by tissue. BGA13: skeletal muscle near ceasarian; BGA14: whole testes; BGA19: mammary gland/parenchyma; BGA22: uterus (intercaruncular); BGA47: frontal cortex; BGA60: abomasum; BGA62: ileum; BGA81: rumen; BGA135: nucleated blood cells; and BGA173: d 90 lactating mammary gland

Differentially methylated cytosines (DMCs) in the CG context have been widely known to play important roles in tissue development while DMCs in non-CGs are not well studied and usually are ignored for their low methylation level in somatic tissues. Here, we merged both the CG and non-CG contexts together, and identified 10,794 DMCs between at least two samples among the ten samples. We found 94.34 % of the DMCs were in the CG context, which supports the predominant role of CG methylations in somatic tissues (Fig. 3a, Additional file 1: Table S3). The DM non-CGs took 5.66 % of the DMCs and were enriched at the high methylation level, which illustrates that differences should be real. There were 4495 DMCs successfully annotated in the regions of 1500 bp upstream of the TSS and gene bodies.

Because RNA-Seq data were generated for eight out of ten tissues (Additional file 1: Table S1), we also generated DMCs derived from only these eight tissues. To detect the effects of a single cytosine methylation on gene expression, we applied Pearson correlation analysis to compare DMCs and RNA-Seq results from these eight shared tissues. We ultimately obtained 3181 cytosines overlapped with 793 genes having both data for correlation analysis. We found that DMCs were divided into two types: 1) DMCs located within 1500 bp upstream of the TSS and enriched in negative correlation with gene expression, and 2) DMCs in the gene body regions showing no obvious correlation preference (Fig. 3b). Totally, there were 408 DMC methylation levels which were significantly (FDR corrected < 0.05) correlated with 117 gene expression levels, and 77.5 % of DMCs showed significant negative correlation (Additional file 1: Table S4). Among all the significant DMCs, 14 non-CG contexts were significantly correlated with gene expression. Gene ontology (GO) analysis of those significantly correlated genes showed no significant GO terms, which was consistent with a similar study in pigs [29].

As expected, most of the significantly correlated CGs were clustered in the genome as they had been proven to be highly correlated with each other within a certain genomic interval. Thus we further detected and analyzed the effects of DMIs related to gene expression levels. Similarly, only the CG islands that overlapped by at least 1 bp with the regions of 1500 bp upstream of the TSS and gene bodies were kept for analysis. In total, we found 836 DMIs wherein 239 of them overlapped with genes that had RNA-Seq information (Additional file 1: Table S5). We found 31 DMIs showed significant correlation with gene expression (Additional file 1: Table S6).

To further evaluate tissue-specific methylation, we considered the DMCs and DMIs in one tissue that appeared different from all other tissues. We detected 798 tissue-specific DMCs (tDMCs) including 75 non-CG tDMCs and 131 tissue-specific DMIs (tDMIs) (Additional file 1: Tables S7, S8). Among the ten samples, the testis (BGA14) displayed the highest counts of tDMCs and tDMIs, which was supported by our clustering results based on DNA methylation patterns (Fig. 3c, Additional file 2: Figure S9) and the previous Bovine Gene Altas study at the transcriptome level [37]. Moreover, we checked the tDMIs whose methylation levels were significantly correlated with gene expression levels and found that all of them belonged to testis. Almost all the testis-specific DMIs showed lower methylation levels than other somatic tissues. Certain “testis-specific antigen” genes, which contain CGIs not methylated in testis but methylated in all other somatic tissues, have been reported to be expressed only in testis [44]. One of the significantly correlated genes, bta-mir-202, was reported to be only expressed in testis and ovary of cattle [45]. Here, we also found it to be highly expressed in testis tissue but not in all other tissues. The average methylation level of the CG island was 52.11 % in BGA14 while in all the other tissues, the methylation levels ranged from 88.10 to 95.10 % (Fig. 3d). Thus, our results supported the negative correlation between a reduced CG island methylation and an increased expression of bta-mir-202 in the testis.

## Discussion

In this study, we constructed DNA methylation profiles of bovine somatic tissues at a single-base resolution using RRBS to provide foundational information for improving our understanding in this area. We found methylation patterns of cattle were similar to those of other species. For example, the mitochondrial genome was comparatively less methylated than the nuclear genome, and the repetitive sequences were highly methylated. The global CG methylation levels detected ranged from 29.87 to 38.06 % among the ten diverse cattle tissues sampled, which were lower than data from pig using RRBS (approximately 40–50 %) [29, 30]. Additionally, a previous study of cattle placenta using WGBS showed the lowest methylation level among all of the mammals they compared [32]. It should be noted that the global methylation level reported by RRBS largely depends on the fraction of DNA methylation within the subset of the genome assessed. The CG island was generally less methylated than the non-CG island [42]. RRBS focuses on the CG-enriched regions which are mostly located in the CG islands [17, 34]. Therefore, the global methylation level reported by RRBS is largely determined by the ratio of detected CGs in CGI regions and non-CG island regions. It is important to point out that RRBS only reports on a small subset of the genome, and more extensive studies like WGBS are needed to confirm these initial RRBS results.

Among the three DNA methylation contexts, CG undoubtedly plays the dominant role in mammals [1]. In the cattle genome, the CG context was the primary contributor to DNA methylation and comprised over 90 % of the DMCs. Among the cytosines detected, 75 % belonged to non-CG contexts which had long been recognized as rarely methylated in mammalian somatic tissues (Fig. 1b). We found that over 10 % of possible cytosine positions within non-CG contexts could be detected as methylated nonredundantly by count, but they were mainly enriched at a low methylation level in cattle somatic tissues. During early embryo development, mammalian genomes undergo a few waves of nearly complete demethylation and remethylation, and DNA methylation statuses differ across tissues and developmental stages [46, 47]. In cattle, the non-CG methylation levels in ten somatic tissues were lower than that in oocytes. We failed to find that non-CG methylation was correlated with the CG methylation in somatic tissues. It is possible that due to the low methylation level of the non-CG, we could not detect changes as observed for the CG methylation levels in the genic and CG island intervals. It is also noted that complete 100 % bisulfite conversion is difficult to achieve without severely degrading DNA. Our data could overestimate non-CG methylation levels and therefore should be treated with caution when used as a reference in future studies. In a pig methylation study, lower methylation was similarly found at the TSS and 5′ end of the gene, however, no obvious methylation difference was found between gene body and non-gene body [30]. The standard model for DNA methylation in mammals is that *de novo* methyltransferases DNMT3a/3b establish the methy-CG landscape in the genome and DNMT1 maintains the CG methylation from the parental strand to the daughter strand at replication forks [20, 21]. However, unlike the CGs, a non-CG motif does not always have a symmetric corresponding non-CG counterpart on the other strand. The proposed “two-step” model cannot fully explain non-CG methylation [10, 22]. Therefore, the non-CG might be mediated by a distinct mechanism as compared to the CGs.

DNA methylation is important for gene expression and plays a critical role in tissue-specific processes [48]. Previous studies focused on the CG context and, thus, the function of non-CG methylation remains unclear [14]. Even though the methylated non-CGs were sparsely distributed within the cattle genome and the global methylation level was low, there were some non-CGs with high methylation levels and differential methylation among tissues. Here, we included the non-CG context when we examined the DMCs. Among the DMCs, we found 611 sites belonging to non-CG context. Correlation analysis also detected 14 non-CG methylations that were significantly associated with gene expression. This implied that the non-CG methylation, along with the CG methylation, may participate in regulating tissue development in cattle. Besides DMCs, we also detected DMIs because most of the differentially methylated CGs were clustered and showed similar distribution among the ten diverse tissues. In the promoter regions, DNA methylation is associated with gene silencing while its function in gene bodies is still controversial [38, 49, 50]. This was supported by our results in which DNA methylation in the upstream 1500-bp regions of TSS showed largely negative correlation with gene expression, while DNA methylation in gene bodies showed a mixed trend. Additionally, a large percentage of DMCs and DMIs were far away from annotated genes. This does not mean that they did not contribute to the tissue differences. A minor reason for this observation may be related to incomplete gene annotation in the cattle genome. Several previous studies support the so-called “orphan CGIs” exhibiting a high degree of tissue-specific methylation regulating gene expression indirectly [51]. Thus our result provided a rich data set of DMCs and DMIs potentially involved in cattle tissue development. It is important to note that due to low methylation levels in the non-CG context (1 to 2 %) and incomplete bisulfite conversion rates (0.45 to 0.97 %), our result and conclusion about methylation in non-CG contexts should be interpreted with caution. Future WGBS experiments with deep coverage are warranted.

## Conclusions

In summary, this study provided baseline methylation profiles for selected cattle genomic regions at a single-base resolution. We characterized the DNA methylome and assessed DNA methylation patterns in ten diverse cattle somatic tissues. We reported many DMCs and DMIs across different tissues and detected a subset correlated with gene expressions. Our study contributes to the understanding of cattle DNA methylation patterns and provides foundational information for further investigations.

## Methods

### Tissues and data collection

The tissues were snap frozen in liquid N_2_ immediately after excision and kept at −80 °C until use. We selected ten tissues including skeletal muscle near ceasarian, whole testes, mammary gland/parenchyma, uterus (intercaruncular), frontal cortex, abomasum, ileum, rumen, nucleated blood cells and d 90 lactating mammary gland (Additional file 1: Table S1). They were coded as BGA13, BGA14, BGA19, BGA22, BGA47, BGA60, BGA62, BGA81, BGA135 and BGA173, respectively, according to the previous Bovine Gene Altas study [37]. The WGBS data for cattle oocyte were downloaded from NCBI GEO dataset under accession number GSE63330. Using a similar collection of tissues as described by Harhay et al. [37], RNA-Seq data were generated on the Illumina HiSeq2000 platform (Illumina, San Diego, CA) using the single end (SE) 100 chemistry. RNA-Seq datasets (at least 2 Gb each) for eight of the ten selected tissues were used for further analysis (Table 1).

### Library construction and sequencing

Genomic DNA for each tissue was isolated according to the QIAamp DNA Mini Kit protocol (QIAGEN, Valencia, CA). RRBS libraries were constructed according to the manufacturer’s instructions. In detail, 3 μg of genomic DNA was digested with the methyl insensitive *MspI* enzyme (CCGG site) at 37 °C for 16 h for each sample. The digested DNA products were purified using the QIAquick PCR Purification Kit (QIAGEN) and single A nucleotides were added to the blunt-end, which were then ligated to a methylated adapter with T overhangs. Ligated products corresponding to DNA fragments 150–400 bp long were isolated and purified using 2.5 % agarose gel electrophoresis. The recovered DNA was treated with the EZ DNA Methylation-Gold Kit (Zymo Research Corp., Irvine, CA) for the bisulfite conversion. DNA with known methylation level was used as a spike control, and all conversion rates were >99 %, ranging from 99.07 to 99.45 % (Table 1). The bisulfite-converted DNA was finally amplified by PCR to construct the RRBS libraries. The Agilent 2100 bioanalyzer instrument (Agilent DNA 1000 Reagents, Agilent, Santa Clara, CA) and real-time quantitative PCR (qPCR, TaqMan Probe) were used to quantitate and quantify the RRBS libraries, respectively. The qualified libraries were amplified on cBot to generate the cluster on the flowcell (TruSeq PE Cluster Kit V3-cBot-HS, Illumina). The HiSeq 2000 system (Illumina) was uses for paired-end sequencing with a 49-bp read length.

### Reads alignment and bioinformatics analysis

Raw sequencing data were processed by an Illumina base-calling pipeline. Raw reads were trimmed for Q score of 20 as the minimum, removing the adapter sequences and multiple N reads. Clean reads were then aligned to the modified cattle reference genome (UMD3.1) by a modified pipeline based on SOAPaligner (version 2.21) in BGI-Shenzhen (Shenzhen, China) as describe previously [52–54]. This modified pipeline ignores all C to T conversions induced by bisulfite treatment and uses three nucleotides alignment strategy and is similar to other bisulphite sequencing alignment software. Only the cytosines with at least eight reads coverage were used for further analysis. We used R (version 3.1.1) script to perform the following statistical analysis [55] . The methylation level of each cytosine site was calculated as the percentage of methylated cytosines to the total cytosines. The methylation levels of each genome feature were defined as the average methylation level of all the annotated cytosines. Only the genes in the RefSeq database were used in the methylation analysis of genic regions to improve the accurate of the genic methylation evaluation. For DMIs, we only kept the CG islands with five or more detected CG sites. We then used the average value of these detected CG sites to represent the whole CG island’s methylation level. The methylKit R package was used to detect DMCs and DMIs with cutoff value of 25 % methylation difference (*q*-value < 0.01) [56]. GO analysis was performed by using the protein IDs to quarry gene ontology terms in AgriGo website software with Fisher’s exact test (http://bioinfo.cau.edu.cn/agriGO/) [57].

### PCR-Sanger sequencing validations of the RRBS results

We performed experimental validations of RRBS results for 66 CG sites distributed in four tissues (whole testes, frontal cortex, ileum and rumen). The primer information can be seen in Additional file 1: Table S9. Three PCR primer pairs were designed using MethPrimer (http://www.urogene.org/cgi-bin/methprimer). The genomic DNA was treated with the EZ DNA Methylation-Gold Kit (Zymo Research Corp.) to apply for bisulfite conversion. PCR was performed in a 25-μl reaction volume according to the Taq DNA polymerase manufacturer’s instructions (QIAGEN instruction (QIGEN, Taq PCR Master Mix Kit). PCR products were purified using QIAquick PCR Purification Kit (QIAGEN) and cloned into T-vector, which was then transformed into *E. coli*. We selected approximately 20 single clones for each PCR product for Sanger sequencing.

### RNA-Seq data and WGBS data analysis

All the collected raw data (RNA-seq and WGBS) were filtered for removing the adapter sequences, contamination and low-quality reads, and the clean reads were aligned to the modified cattle reference genome (UMD3.1). For RNA-Seq data, we applied Tophat (Version 2.0.13) and Cufflink (Version 2.2.1) protocols according to the previously published paper using the default parameters [58]. For WGBS data of oocytes, we aligned the clean reads on the two modified references with Bismark (Version 0.14.5) using Bowtie 2 which allowed no mismatch [59]. Only uniquely aligned reads were used to determine the methylation status. The methylation status were extracted using the Bismark methylation extractor with optional genome-wide cytosine report output.

## Additional files

Additional file 1: Table S1. Tissue samples used in the RRBS analysis. **Table S2.** RRBS validation results. **Table S3.** Different methylated cytosine information. **Table S4.** Significantly correlated DMCs and gene information. **Table S5.** Different methylated CpG island information. **Table S6.** Significantly correlated DMIs and gene information. **Table S7.** Tissue-specific different methylated cytosine information. **Table S8.** Tissue-specific different methylated CpG island information. **Table S9.** Primers used for validation of RRBS results. (XLS 2962 kb)
Additional file 2: Figure S1. Distribution for the percentage of cytosine with 2 to 11 reads. **Figure S2.** Methylation of different methylation contexts for cattle somatic tissues. (a) CG percentages with different methylation levels; (b) CHG percentages with different methylation levels; (c) CHH percentages with different methylation levels. Note: the error bar represents the standard deviation among the 10 tissues. **Figure S3.** Correlation analysis of CG and non-CG methylation using 1-Mb non-overlapping windows for oocyte overlapped with the RRBS data. Note: Only the cytosines that overlapped with the RRBS data in oocyte WGBS were used for plotting. **Figure S4.** Methylation levels for different genomes. Note: the error bar represents the standard deviation among the 10 tissues. **Figure S5.** Methylation levels for different repetitive sequences. Note: the error bar represents the standard deviation among the 10 tissues. **Figure S6.** Methylation distributions of the 3 methylation contexts in genic regions and CG islands for oocyte overlapped with the RRBS data. **Figure S7.** Autocorrelation analysis for different methylation contexts on genome. Chr1 was used to calculate the correlation of different methylation contexts with different distances. Note: all figures for somatic tissues were from the merged data after examining results individually that did not show differences between them. **Figure S8**. CG island methylations in cattle somatic tissues. (a) Average methylation levels of CG islands, CG island shores and non-CG island regions. (b) Distribution of CG islands at different methylation levels. (c) CG island methylation levels within every window of 10 % length of all chromosomes. The x-axis is the interval of all chromosomes from 5′ to 3′. **Figure S9.** Clustering of 10 tissues based on 131 tissue-specific DMIs (tDMIs). (PDF 896 kb)

## Abbreviations

DMC: Differentially methylated cytosine
DMI: Differentially methylated CG island
FAANG: The Functional Annotation of Animal Genome project
GO: Gene ontology
LINE: Long interspersed nuclear element
LTR: Long terminal repeat
MBD-seq: Methyl-binding domain sequencing
MeDIP-seq: Methylated DNA immunoprecipitation combined with high-throughput sequencing
RRBS: Reduced representation bisulphite sequencing
SINE: Long interspersed nuclear element
tDMC: tissue-specific DMC
tDMI: tissue-specific DMI
TSS: Transcription start site
WGBS: Whole genome bisulfite sequencing
