# Supplementary material for: Reduced representation bisulphite sequencing of ten bovine somatic tissues reveals DNA methylation patterns and their impacts on gene expression
Source: BMC Genomics. 2016 Oct 6;17:779. doi: 10.1186/s12864-016-3116-1 (PMC5053184; doi:10.1186/s12864-016-3116-1)
Supplement: Additional file 2: Figure S1. — Distribution for the percentage of cytosine with 2 to 11 reads. Figure S2. Methylation of different methylation contexts for cattle somatic tissues. (a) CG percentages with different methylation levels; (b) CHG percentages with different methylation levels; (c) CHH percentages with different methylation levels. Note: the error bar represents the standard deviation among the 10 tissues. Figure S3. Correlation analysis of CG and non-CG methylation using 1-Mb non-overlapping windows for oocyte overlapped with the RRBS data. Note: Only the cytosines that overlapped with the RRBS data in oocyte WGBS were used for plotting. Figure S4. Methylation levels for different genomes. Note: the error bar represents the standard deviation among the 10 tissues. Figure S5. Methylation levels for different repetitive sequences. Note: the error bar represents the standard deviation among the 10 tissues. Figure S6. Methylation distributions of the 3 methylation contexts in genic regions and CG islands for oocyte overlapped with the RRBS data. Figure S7. Autocorrelation analysis for different methylation contexts on genome. Chr1 was used to calculate the correlation of different methylation contexts with different distances. Note: all figures for somatic tissues were from the merged data after examining results individually that did not show differences between them. Figure S8. CG island methylations in cattle somatic tissues. (a) Average methylation levels of CG islands, CG island shores and non-CG island regions. (b) Distribution of CG islands at different methylation levels. (c) CG island methylation levels within every window of 10 % length of all chromosomes. The x-axis is the interval of all chromosomes from 5′ to 3′. Figure S9. Clustering of 10 tissues based on 131 tissue-specific DMIs (tDMIs). (PDF 896 kb) [file 12864_2016_3116_MOESM2_ESM.pdf]

Figure S1. Distribution for the percentage of cytosine with 2 to 11 reads.

Figure S2. Methylation of different methylation contexts for cattle somatic tissues. (a) CG percentages with different methylation levels; (b) CHG percentages with different methylation levels; (c) CHH percentages with different methylation levels. Note: the error bar represents the standard deviation among the 10 tissues.

Figure S3. Correlation analysis of CG and non-CG methylation using 1-Mb non-overlapping windows for oocyte overlapped with the RRBS data. Note: Only the cytosines that overlapped with the RRBS data in oocyte WGBS were used for plotting.

Figure S4. Methylation levels for different genomes. Note: the error bar represents the standard deviation among the 10 tissues.

Figure S5. Methylation levels for different repetitive sequences. Note: the error bar represents the standard deviation among the 10 tissues.

Figure S6. Methylation distributions of the 3 methylation contexts in genic regions and CG islands for oocyte overlapped with the RRBS data.

Figure S7. Autocorrelation analysis for different methylation contexts on genome. Chr1 was used to calculate the correlation of different methylation contexts with different distances. Note: all figures for somatic tissues were from the merged data after examining results individually that did not show differences between them.

Figure S8. CG island methylations in cattle somatic tissues. (a) Average methylation levels of CG islands, CG island shores and non-CG island regions. (b) Distribution of CG islands at different methylation levels. (c) CG island methylation levels within every window of 10% length of all chromosomes. The x-axis is the interval of all chromosomes from 5’ to 3’.

Figure S9. Clustering of 10 tissues based on 131 tissue-specific DMIs (tDMIs).

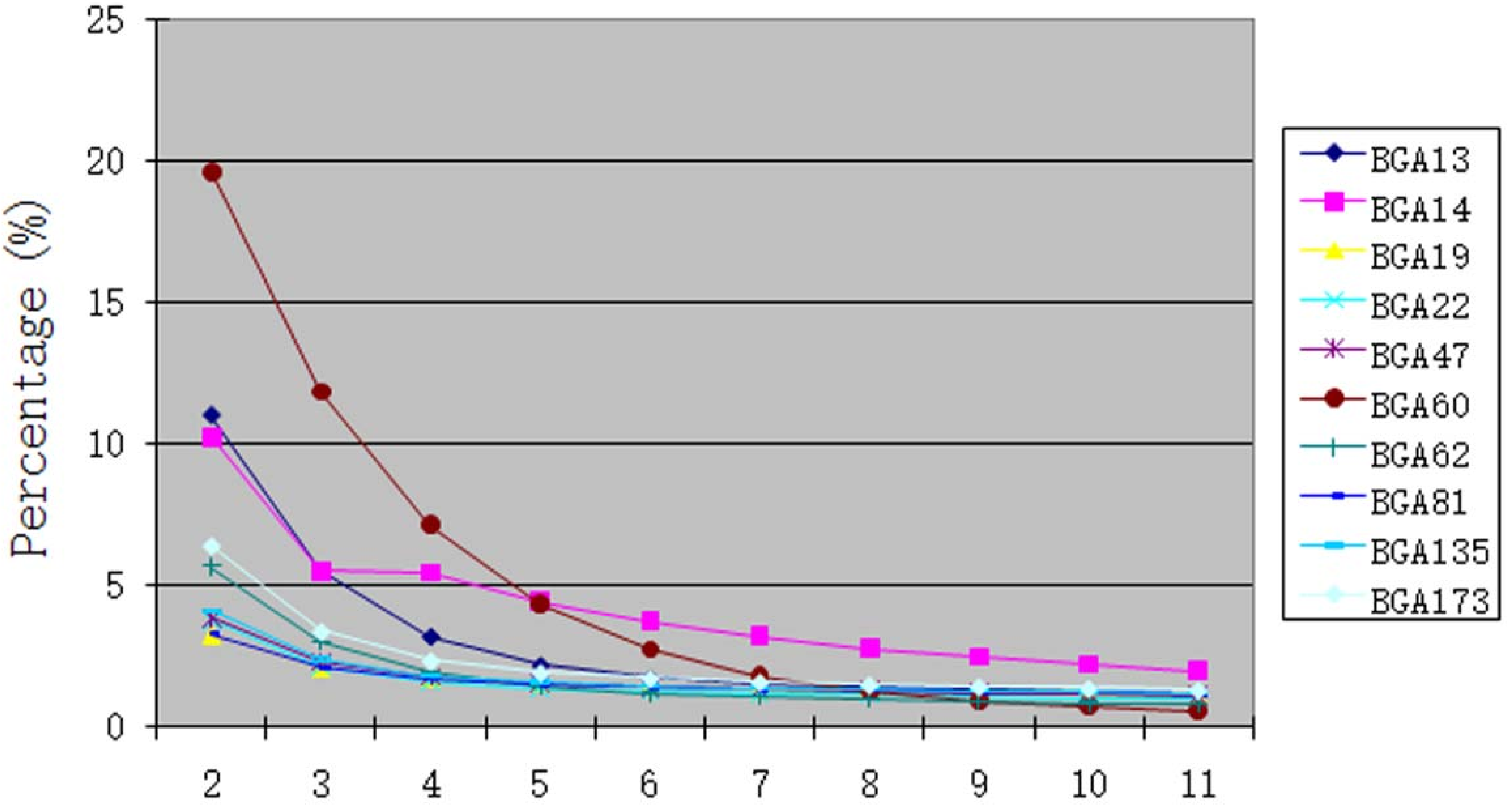

Percentage (%)
25
20
15
10
5
0
2
3
4
5
6
7
8
9
10
11
BGA13
BGA14
BGA19
BGA22
BGA47
BGA60
BGA62
BGA81
BGA135
BGA173

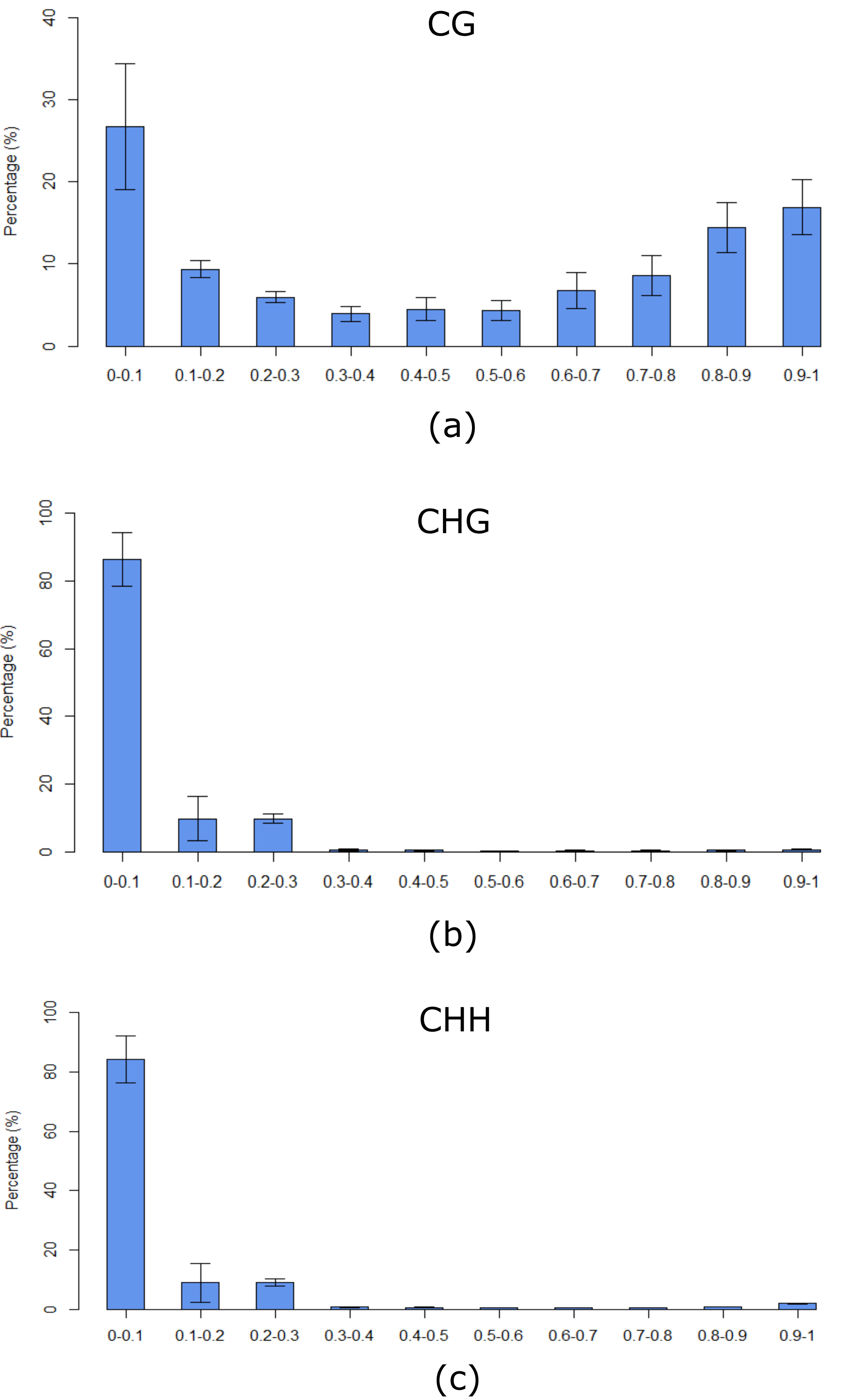
CG
Percentage (%)
0
10
20
30
40
0-0.1
0.1-0.2
0.2-0.3
0.3-0.4
0.4-0.5
0.5-0.6
0.6-0.7
0.7-0.8
0.8-0.9
0.9-1
(a)
CHG
Percentage (%)
0
20
40
60
80
100
0-0.1
0.1-0.2
0.2-0.3
0.3-0.4
0.4-0.5
0.5-0.6
0.6-0.7
0.7-0.8
0.8-0.9
0.9-1
(b)
CHH
Percentage (%)
0
20
40
60
80
100
0-0.1
0.1-0.2
0.2-0.3
0.3-0.4
0.4-0.5
0.5-0.6
0.6-0.7
0.7-0.8
0.8-0.9
0.9-1
(c)

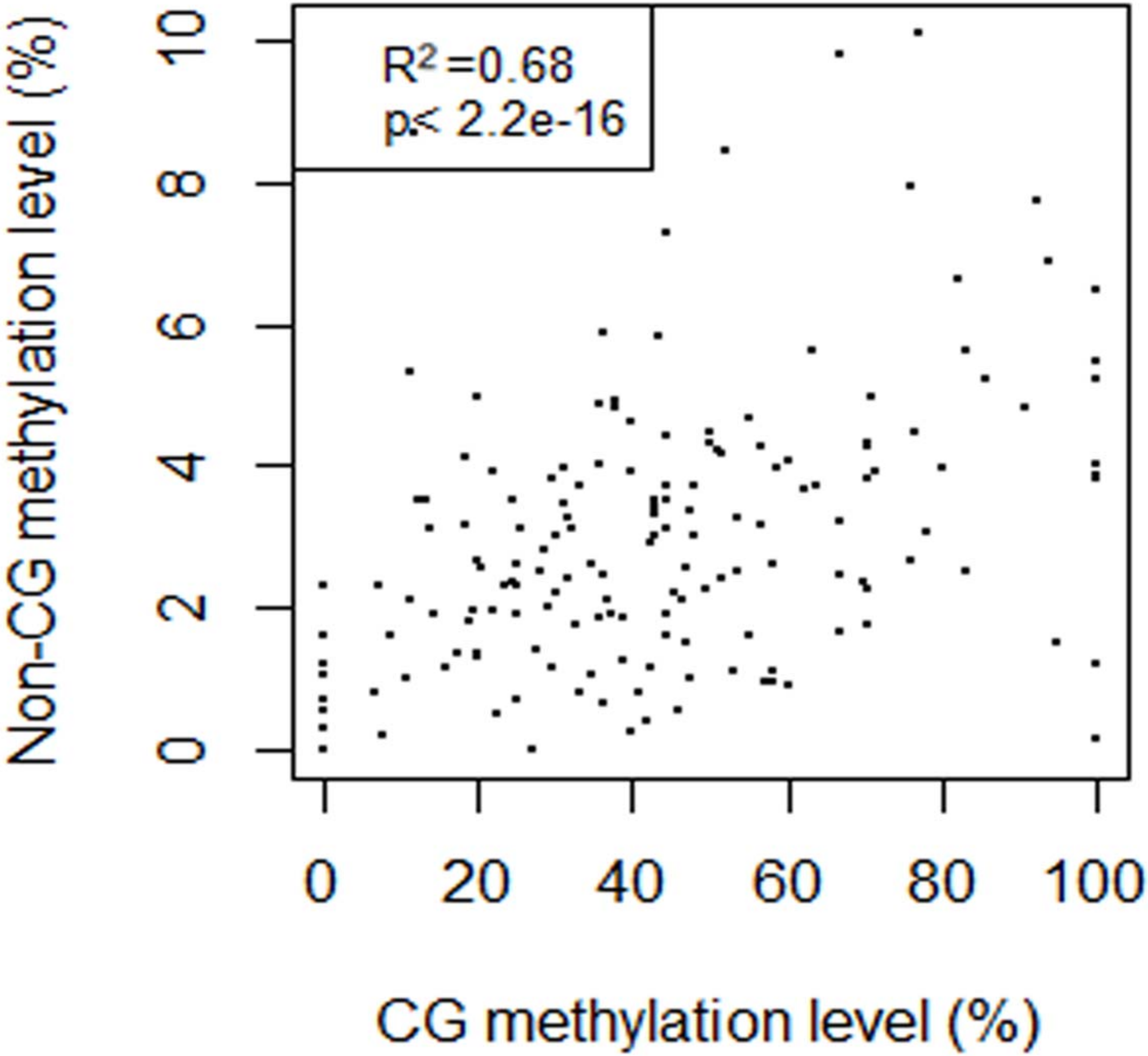

R^2 =0.68
p< 2.2e-16
Non-CG methylation level (%)
0
2
4
6
8
10
0
20
40
60
80
100
CG methylation level (%)

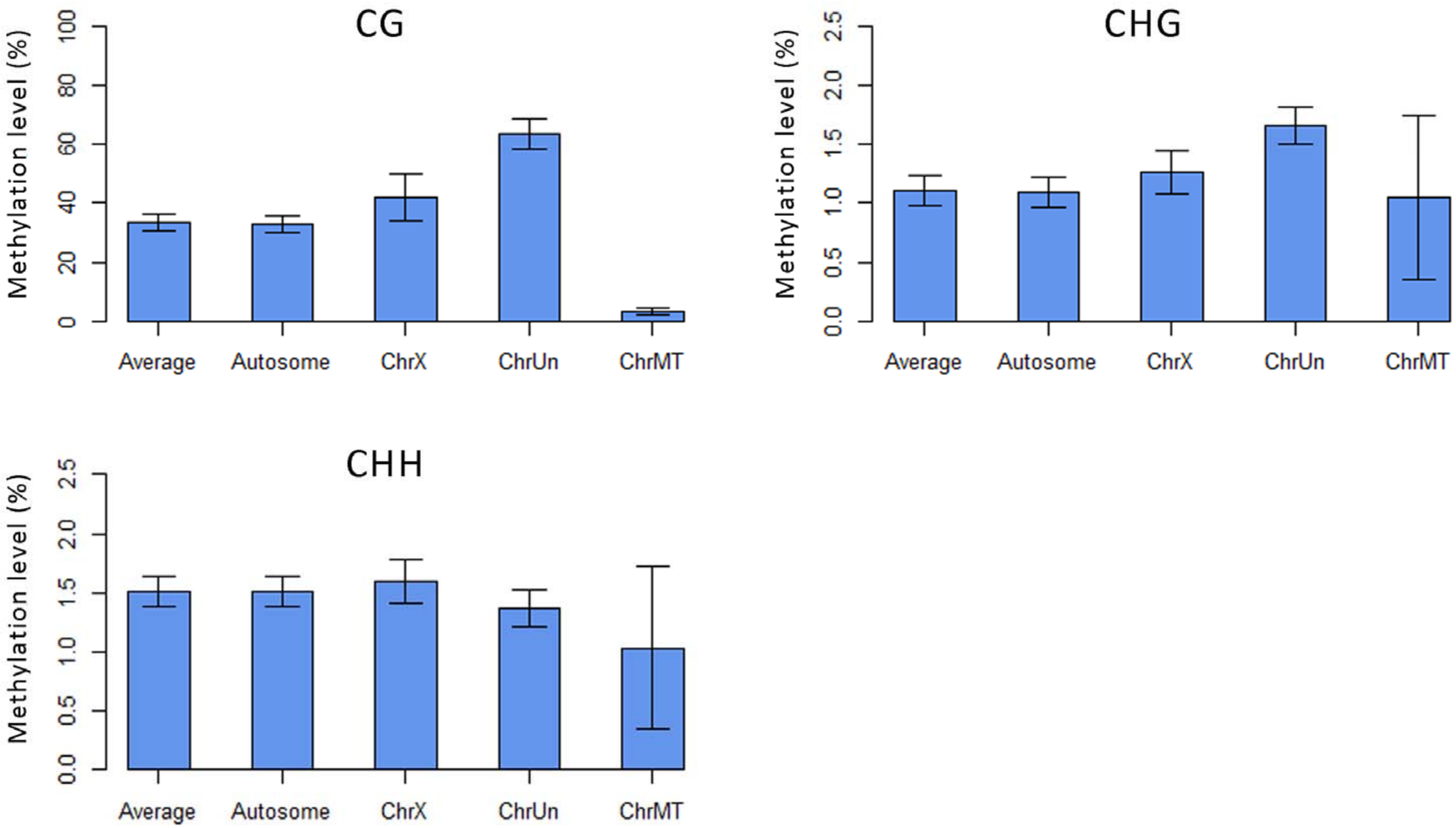
CG
CHG
CHH
Methylation level (%)
0
20
40
60
80
100
0.0
0.5
1.0
1.5
2.0
2.5
Average
Autosome
ChrX
ChrUn
ChrMT

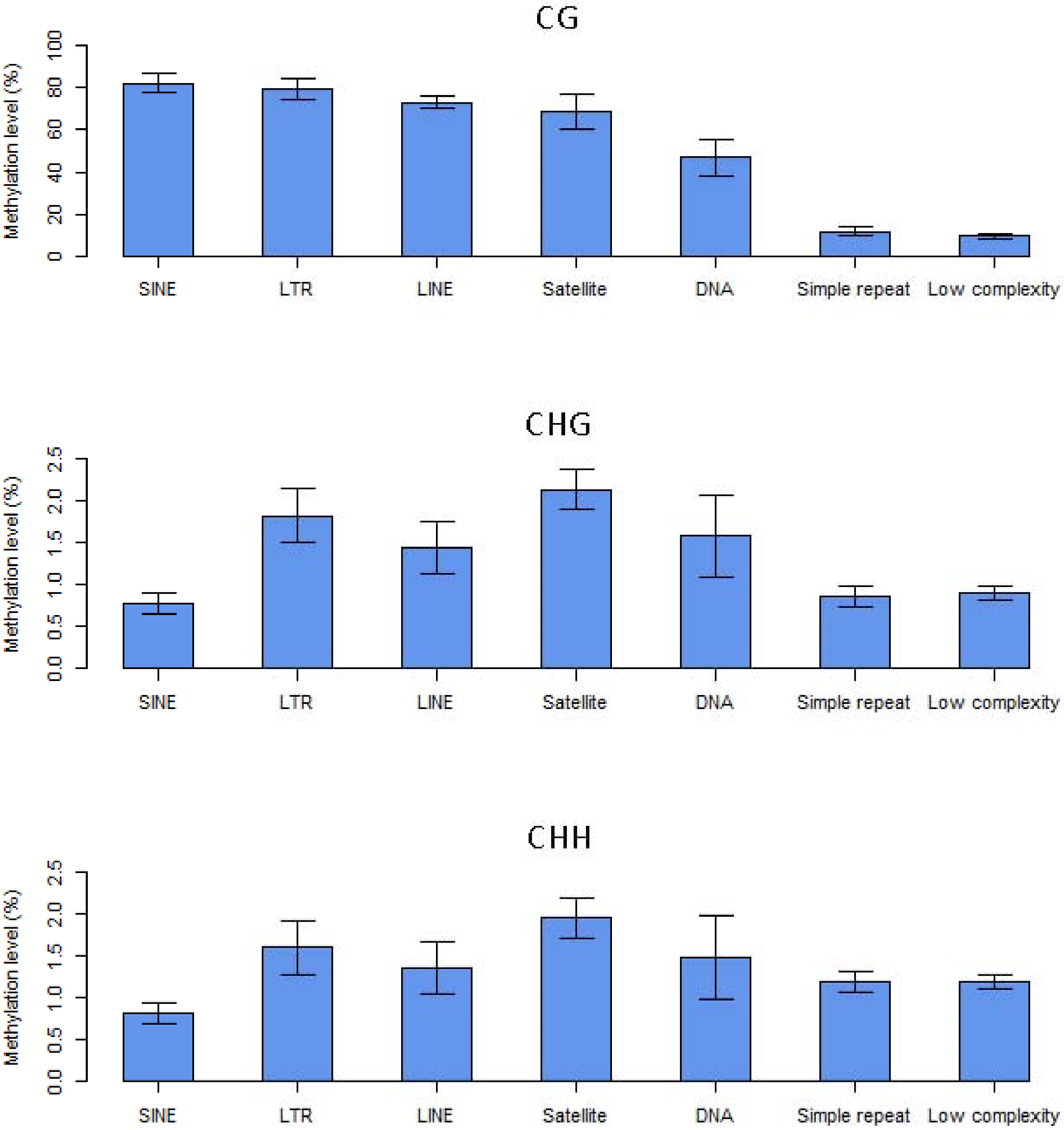
CG
Methylation level (%)
0
20
40
60
80
100
SINE
LTR
LINE
Satellite
DNA
Simple repeat
Low complexity
CHG
Methylation level (%)
0.0
0.5
1.0
1.5
2.0
2.5
SINE
LTR
LINE
Satellite
DNA
Simple repeat
Low complexity
CHH
Methylation level (%)
0.0
0.5
1.0
1.5
2.0
2.5
SINE
LTR
LINE
Satellite
DNA
Simple repeat
Low complexity

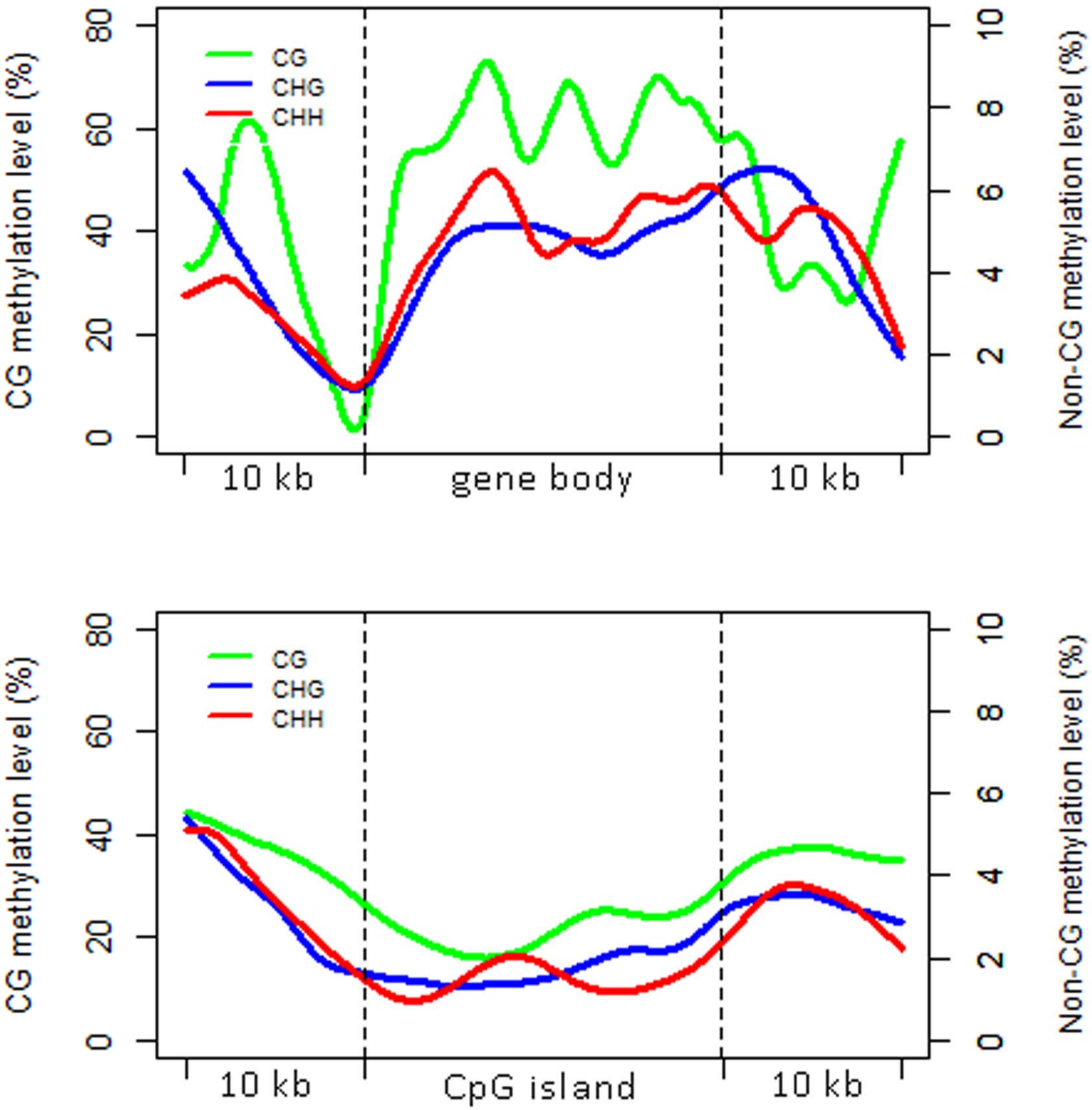
CG
CHG
CHH
CG methylation level (%)
Non-CG methylation level (%)
0
20
40
60
80
0
2
4
6
8
10
10 kb
gene body
10 kb
CG
CHG
CHH
CG methylation level (%)
Non-CG methylation level (%)
0
20
40
60
80
0
2
4
6
8
10
10 kb
CpG island
10 kb

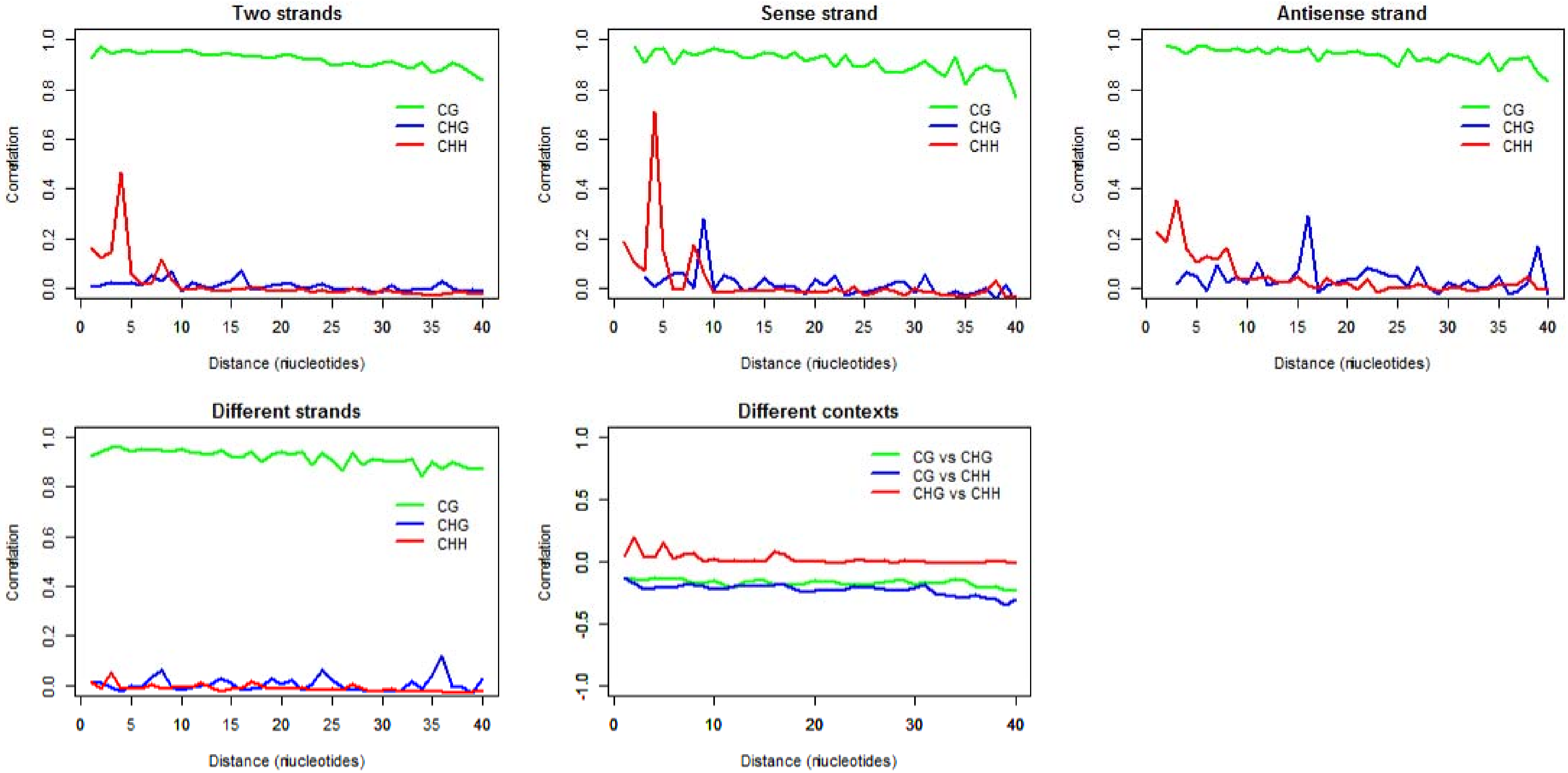
Two strands
Sense strand
Antisense strand
Different strands
Different contexts
CG
CHG
CHH
CG vs CHG
CG vs CHH
CHG vs CHH
Correlation
Distance (nucleotides)

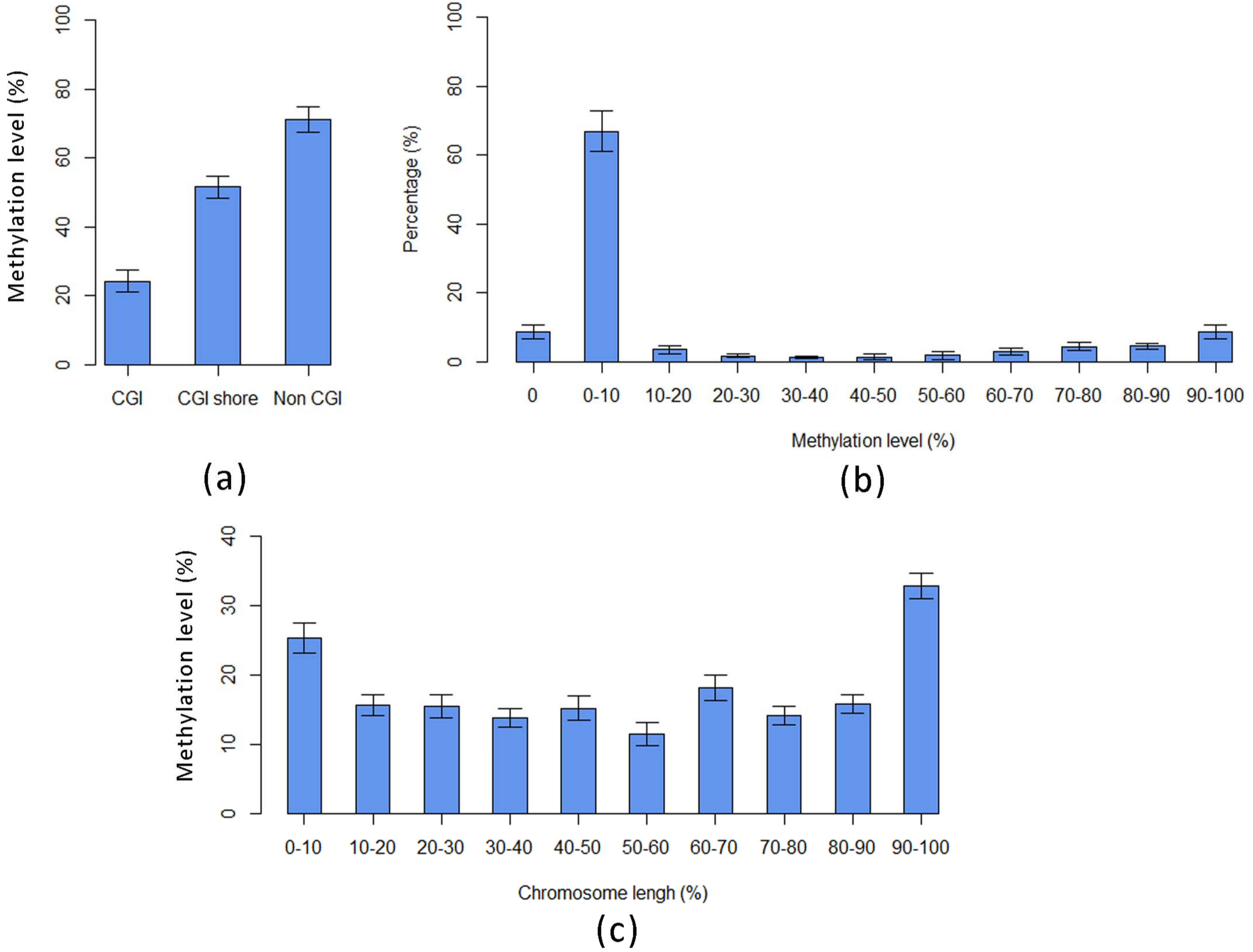
Methylation level (%)
0
20
40
60
80
100
CGI
CGI shore
Non CGI
(a)
Percentage (%)
0
20
40
60
80
100
0
0-10
10-20
20-30
30-40
40-50
50-60
60-70
70-80
80-90
90-100
Methylation level (%)
(b)
Methylation level (%)
0
10
20
30
40
0-10
10-20
20-30
30-40
40-50
50-60
60-70
70-80
80-90
90-100
Chromosome lengh (%)
(c)

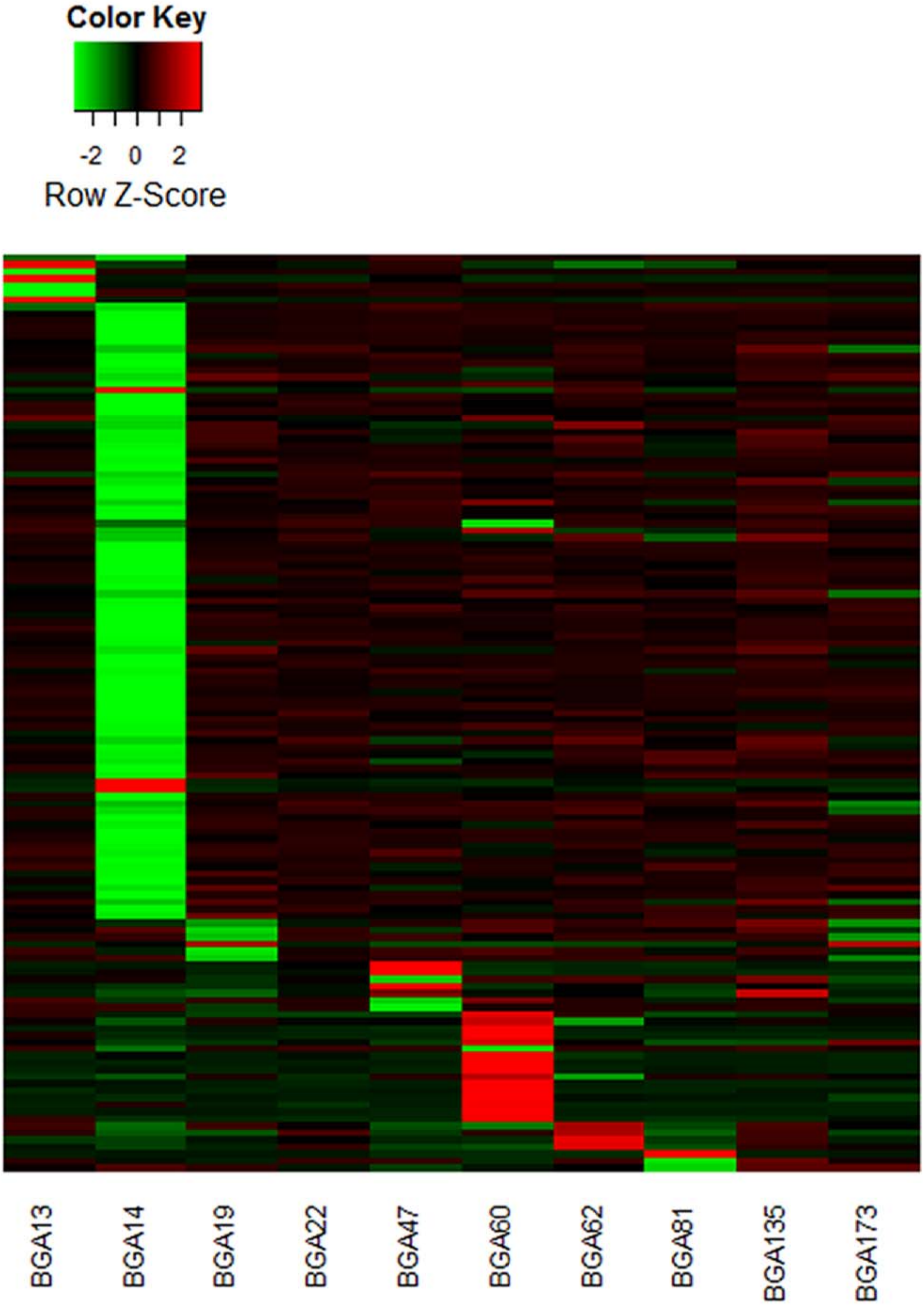
Color Key
-2 0 2
Row Z-Score
BGA13
BGA14
BGA19
BGA22
BGA47
BGA60
BGA62
BGA81
BGA135
BGA173
